# Effects of temperature and drought stress on the seed germination of a peatland lily (*Lilium concolor* var. *megalanthum*)

**DOI:** 10.3389/fpls.2024.1462655

**Published:** 2024-10-11

**Authors:** Mingfan Guo, Jing Zong, Jinxin Zhang, Li Wei, Wenguang Wei, Rongyang Fan, Tingting Zhang, Zhanhui Tang, Gang Zhang

**Affiliations:** ^1^ China Energy Engineering Group Guangxi Electric Power Design Institute CO., LTD, Nanning, China; ^2^ State Environmental Protection Key Laboratory of Wetland Ecology and Vegetation Restoration, Northeast Normal University, Changchun, China; ^3^ China Power Engineering Consulting Group CO., LTD, Beijing, China; ^4^ Technical Innovation Center of Mine Geological Environmental Restoration Engineering in Southern Karst Area, Ministry of Natural Resources, Nanning, China; ^5^ School of Environment, Northeast Normal University, Changchun, China; ^6^ Key Laboratory of Vegetation Ecology, Ministry of Education, Northeast Normal University, Changchun, China; ^7^ Institute of Grassland Science, Northeast Normal University, Changchun, China

**Keywords:** *Lilium concolor* var. *megalanthum*, temperature, drought stress, interaction, seed germination

## Abstract

Sexual reproduction through seeds is an effective way to renew plant populations and increase their genetic diversity, but seed germination process is complicated and relatively difficult due to the restriction of environmental conditions. Wetland plants that reproduce sexually through seeds may be affected by changes in moisture and temperature. This study aims to explore the ecological adaptation strategies of seed germination of *Lilium concolor* var. *megalanthum* under different hydrothermal conditions. Controlled experiments were conducted to investigate the germination performance of *L. concolor* var. *megalanthum* seeds at different temperatures (10°C, 15°C, 20°C, 25°C, and 30°C) and simulated drought stress conditions using PEG-6000 solutions (0%, 5%, 10%, 15%, and 20%). The results showed that temperature, drought stress, and their interaction significantly affected the days to first germination, germination percentage, coefficient of germination rate, germination energy, germination index, and vigor index of seeds (*p*<0.01). The germination percentage, germination index, and vigor index of seed were significantly higher at 25°C compared to other temperatures (*p*<0.01). The interaction between low temperature and drought stress significantly delayed the days to first germination. The inhibition of drought stress on seed germination was enhanced by PEG-6000 solution under high temperature. Under the conditions of 25°C and 5% PEG-6000 solution concentration, seeds of *L. concolor* var. *megalanthum* exhibited optimal germination parameters. At 10°C and 15°C, the seeds exhibited the highest tolerance to PEG-6000-simulated drought stress. Rehydration germination results showed that extreme temperatures and drought stress conditions inhibit seed germination of *L. concolor* var. *megalanthum* without damaging seed structure. The germination pattern of seeds under variable temperature and drought stress conditions reflects their adaptive strategies developed over long-term evolution to cope with the environmental conditions.

## 1 Introduction

Seed germination is a crucial stage in the life history of flowering plants, influenced by both seed characteristics (such as coat thickness and dormancy) and external environmental conditions. This stage is particularly vulnerable and critical for plant population renewal (Sy et al., 2001; Ghaderi-Far et al., 2010). Due to differences in plant species and their habitats, the germination characteristics of plant seeds and their responses to environmental factors vary (Zhang et al., 2020). Therefore, the seed germination process of flowering plants and its influencing factors have become one of the research hotspots in the fields of ecology and botany.

Temperature is a particularly significant ecological factor influencing seed germination (Sun and Li, 2020; Zhang et al., 2020). Temperature affects seed germination by influencing both dormancy release and the germination process itself (Roberts, 1988; Batlla and Benech-Arnold, 2015). Three key temperature thresholds are essential for seed germination: base temperature, optimum temperature, and maximum temperature. Germination parameters, such as germination percentage and coefficient of germination rate, generally increase from the base temperature to the optimum temperature and then decline from the optimum to the maximum temperature (Steinmaus et al., 2000; Ghaderi-Far et al., 2010). The optimum temperature for germination varies among plant species due to differences in individual traits, habitats, and physiological ecology (Kumar et al., 2011). For example, *Carthamus tinctorius* seeds have an optimum germination temperature range of 21.4°C–29°C (Torabi et al., 2016), whereas *Ziziphus lotus* seeds have a higher optimum temperature of 35°C (Maraghni et al., 2010), and *Cuminum syminum* seeds have a lower optimum temperature of 15°C (Rahimi, 2013). Both critical high and low temperatures can affect the membrane permeability of seeds, and the utilization of oxygen and enzymatic activity during germination, thereby restricting the germination process (Baskin and Baskin, 1988; Bewley and Black, 1994; Verma et al., 2010).

Moisture condition is also an important factor affecting seed germination. Typically, moisture absorption by seeds is usually divided into three stages. In the initial stage, seeds absorb water to meet the seed matrix potential (Kebreab and Murdoch, 1999; Allen, 2003); in the mid-stage, viable seeds undergo physiological metabolism in preparation for radical emergence (Bewley and Black, 1994); in the final stage, the water absorption rate increases sharply, and radicles begin to emerge and grow. Different plant seeds have varying moisture requirements for germination, and seeds can only germinate when moisture conditions exceed the critical threshold required for germination (Lewandrowski et al., 2017).

Global climate change predictions indicate an increase in droughts and heatwave events. Moisture and temperature typically interact in regulating seed germination. Extreme high or low temperatures and low soil water potential can all affect seed germination, potentially restricting plant seedling regeneration and ultimately impacting plant population dynamics (Gurvich et al., 2017). In studies exploring the interactive effects of temperature and drought stress on seed germination, research by Guedes et al. indicates that *Apeiba tibourbou* seed germination is significantly affected by the interaction of temperature and drought stress. The inhibition of seed germination under PEG-6000 stress is mainly attributed to the low water potential caused by osmosis effects. Under water potential of −0.2 MPa, the seed germination percentage markedly decreases, with a germination percentage of 51% at 30°C and the most pronounced reduction to 37% at 25°C (Guedes et al., 2013). Silva et al. (2014) found that the germination percentage of *Barbarea verna* seeds decreases with decreasing water potential and that the higher temperature (35°C) exhibits a stronger inhibitory effect on germination compared to lower temperatures (20°C and 25°C). Most seeds that fail to germinate under PEG-simulated drought conditions remain viable, and they can germinate promptly and relatively uniformly when drought stress was relieved (Lin et al., 2016; Jose Vicente et al., 2020). This adaptive behavior represents a survival strategy for plants in adverse environment. For instance, Elnaggar et al. (2018) found that the seed germination of *Salsola imbricata* can tolerate relatively low drought stress, and seeds that fail to germinate under drought stress recover germination faster at lower temperatures than at higher temperatures.

Currently, nearly half of the wetlands worldwide are experiencing drought and degradation, and efforts to restore biodiversity are also challenged by invasive species (Zedler and Kercher, 2005). In particular, inland peatlands are greatly influenced by the changes in hydrological conditions, which affect species diversity and the succession of plant community. Localized temperature changes caused by global climate change can also affect seed germination of wetland plants, thereby influencing their population dynamics. For some species with rare distributions in inland peatlands, they are more susceptible to changes in ecological factors caused by habitat changes. Therefore, it is necessary to study the effects of sensitive ecological factors, such as water and temperature, on the reproductive processes of these plants. As the only lily species occurring in peatlands of the Changbai Mountains in northeastern China, *L. concolor* var. *megalanthum* has a narrow distribution range. Its population is scarce and declining year by year. It can serve as an indicator species for changes in peatlands environments. Exploring the status and processes of its population renewal is of great significance for predicting population dynamics and developing effective species conservation and restoration strategies. *L. concolor* var. *megalanthum* can reproduce and expand its population through sexual reproduction (seeds) and asexual reproduction (bulbs). Due to the decrease in population numbers and density, the availability of bulbs is limited. The identification of seed germination characteristics under temperature and drought stress conditions is highly urgent. However, there are currently no reports on the seed germination characteristics of *L. concolor* var. *megalanthum*. This study aims to investigate the effects of temperature treatments and PEG-6000-simulated drought stress on the germination characteristics of *L. concolor* var. *megalanthum* seeds, revealing the ecological adaptation mechanisms of seed germination to moisture and temperature conditions. The results may provide a theoretical basis for predicting the impact of future global climate change-induced wetland degradation on seed germination and for identifying the optimal temperature and moisture conditions for seed germination.

## 2 Materials and methods

### 2.1 Seed collection

The seeds used for the experiment were collected in late September 2020 from *L. concolor* var. *megalanthum* plants in the Jinchuan peatland of Longwan National Nature Reserve, Tonghua City, Jilin Province, China. The seeds that were plump and uniform in size were selected and stored at room temperature for later use.

### 2.2 Germination experiment and calculation of germination parameters

Five constant temperature conditions (10°C, 15°C, 20°C, 25°C, and 30°C) were established using five environmental chambers (MGC-450HP, Shanghai Yiheng Instruments Co., Ltd., Shanghai, China). Polyethylene glycol 6000 (PEG-6000) is a kind of macromolecular organic compound widely used in drought stress simulation in the study of plant drought resistance due to its inability to penetrate plant cell membrane and cell walls (Carpita et al., 1979; Muscolo et al., 2014; Fakhfakh et al., 2018). PEG-6000 solutions with mass fractions of 5%, 10%, 15%, and 20% were used to simulate various levels of drought stress, with sterile water treatment serving as control treatment (CK). In total, there were 25 treatment combinations, with four replicates assigned to each treatment combination. Each glass Petri dish containing the seed treatment combination was considered a replicate. Plump and intact seeds of uniform size with healthy embryos (where embryo length is ≥ 1/2 seed length) were selected. The seeds were disinfected in a 0.5% KMnO_4_ solution for 8 min, rinsed with sterile water, and dried using a filter paper. A total of 30 seeds were evenly placed in a 90-mm diameter glass Petri dish lined with two layers of filter paper. Then, 8 ml of PEG-6000 solution of corresponding concentration or sterile water was added using a pipette. The Petri dish containing the seeds was placed inside environmental chambers set to different temperatures. The light cycle consisted of 12 h of light (from 6:00 to 18:00) followed by 12 h of darkness, with a light intensity of 5,500 lx. Throughout the experiment, the filter paper was refreshed every 48 h to prevent water evaporation from affecting the concentration of the PEG-6000 solution. The number of germinated seeds in each Petri dish was counted daily. Seed germination was defined as the radicle breaking through the seed coat by at least 2 mm, and the length of the radicle was measured and recorded using a Vernier caliper. Once recorded, germinated seeds were promptly removed. The germination experiment was continuously monitored and recorded over a period of 244 days. The germination parameters and their respective calculation formulas used are outlined below (Yan et al., 2016):


$$\begin{array}{c} \text{Days to first germination }\left(DFG\right):\\ \text{Time from sowing to the germination of the first seed }\left(\text{d}\right) \end{array}$$

$$\begin{array}{c} \text{Germinationpercentage}\left(GP\right)\\ =(\text{number of germinated seeds}/\text{number of tested seeds})\;\times \;100\% \end{array}$$

Coefficient of germination rate (CGR)=[∑​(t×n)/∑​n]×100% , where *t* is the number of days since the beginning of the germination experiment, and *n* refers to the number of seed that germinated within *t* days.


$$\begin{array}{c} \text{Germination energy }\left(GE\right)\\ \;=\;(\text{number of germinated seeds at the maximum germination day}/\\ \text{the number of tested seeds})\;\times \;100\% \end{array}$$

$\text{Germination index }\left(GI\right)=\sum \left(G_{t}/D_{t}\right)$ , where *G_t_* is the number of germinated seeds on day *t*, and *D_t_* is the number of days from sowing to day *t*.

Vigorindex (VI) =GI×lr , where *lr* is the average length of the primary root (cm).

### 2.3 Rehydration test and calculation of germination parameters

After 244 days of the germination experiment, the remaining ungerminated seeds from the aforementioned treatment were washed several times with sterile water and dried using filter paper. Rehydration testing was conducted under the following conditions: 25°C, 12 h light (6:00–18:00)/12h darkness, with a light intensity of 5,500 lx. Aseptic water was used instead of a PEG-6000 solution for rehydration test. Daily observations were conducted, and the experiment was terminated when these ungerminated seeds were monitored for 27 days and there was no seed germination for 14 consecutive days. The germination parameter in the rehydration test and its calculation formula is as follows:


$$\begin{array}{c} \text{Rehydration germination percentage }\\ =\;(\text{number of seeds germinated after rehydration}/\\ \text{total number of seeds rehydrated })\text{that did not }\\ \text{germinated after stress treatment }\\ \times \;100\%. \end{array}$$

### 2.4 Data analysis

The experimental data underwent statistical analysis using SPSS 20.0 and were visualized using Origin 9.2. Two-way ANOVA was employed to examine the effects of temperature, PEG solution concentration, and their interaction on seed germination parameters. Differences between various temperatures and PEG solution concentrations at the same temperature were assessed using the Duncan method of one-way ANOVA analysis. Pearson correlation analysis was used to explore the relationship between PEG solution concentration and germination parameters across different temperature conditions. Statistical significance was set at α = 0.05, and the statistical data are presented as mean ± standard error.

## 3 Results

### 3.1 The effect of temperature on seed germination

Temperature had a significant impact on several seed germination parameters of *L. concolor* var. *megalanthum* (*p*<0.001) (**Table 1**). Days to first germination occurred earlier at 20°C (11.55 days) and 25°C (11.05 days), while higher and lower temperatures caused significant delays (*p<*0.05) (**Table 2**). Germination percentage increased initially and then decreased with rising temperature, peaking at 95.83% at 25°C, which was significantly higher than at other temperatures (*p<*0.01). The coefficient of germination rate decreased gradually with increasing temperature, reaching a minimum at 25°C (28.05), but increased significantly from 25°C to 30°C (*p<*0.01). Germination energy was highest at 20°C (19.00%) and 25°C (18.00%), significantly exceeding other temperatures (*p<*0.01), with no significant difference between 10°C and 15°C (*p>*0.05). The germination index and vigor index were 1.86 and 0.06, respectively, at 25°C, significantly higher than at other temperatures (*p<*0.01). Conversely, these indices were lower at 10°C (0.32 and 0.01) and 15°C (0.37 and 0.01), with no significant difference between them (*p>*0.05).

**Table 1:** Two-way ANOVA analysis of effects of temperature, simulated drought stress of polyethylene glycol (PEG-6000), and their interactions on seed germination parameters of *Lilium concolor* var. *megalanthum*.

		*F*-values					
Factors	df	DFG	GP	CGR	GE	GI	VI
Temperature (T)	4	114.85^***^	5.37^***^	144.36^***^	36.46^***^	472.78^***^	283.67^***^
Drought stress (D)	4	48.08^***^	3.71^***^	48.77^***^	25.96^***^	226.94^***^	169.72^***^
T×D	16	2.06^*^	1.52^***^	1.83^*^	4.80^***^	30.00^***^	25.89^***^

*p<0.05, **p<0.01, ***p<0.001.

DFG, days to first germination; GP, germination percentage; CGR, coefficient of germination rate; GE, germination energy; GI, germination index; VI, vitality index.

**Table 2:** Effects of temperature on the seed germination of *Lilium concolor* var. *Megalanthum*.

Temperature	DFG	GP	CGR	GE	GI	VI
10 (n=20)	38.05 ± 2.61a	71.17 ± 5.28d	101.95 ± 5.66a	8.33 ± 0.82c	0.32 ± 0.03d	0.01 ± 0.00d
15 (n=20)	25.90 ± 2.37b	71.00 ± 5.16d	99.85 ± 5.31a	7.33 ± 0.71c	0.37 ± 0.04d	0.01 ± 0.00d
20 (n=20)	11.55 ± 0.97d	88.67 ± 3.88b	43.30 ± 6.89b	19.00 ± 2.19a	1.68 ± 0.18b	0.05 ± 0.01b
25 (n=20)	11.05 ± 0.81d	95.83 ± 2.08a	28.05 ± 4.62c	18.00 ± 1.57a	1.86 ± 0.18a	0.06 ± 0.01a
30 (n=20)	16.25 ± 2.00c	78.00 ± 5.79c	36.60 ± 3.48b	13.17 ± 1.46b	1.13 ± 0.13c	0.03 ± 0.00c

Different lowercase letters in each column represent significant differences at 0.05 levels.

DFG, days to first germination; GP, germination percentage; CGR, coefficient of germination rate; GE, germination energy; GI, germination index; VI, vitality index.

### 3.2 The effect of drought stress on seed germination

Different concentrations of PEG-6000 solution simulating drought stress had a significant effect on the days to first germination, germination percentage, coefficient of germination rate, germination energy, germination index, and vigor index of *L. concolor* var. *megalanthum* seeds (*p<*0.001) (**Table 1**). The days to first germination were significantly delayed at 15% and 20% PEG-6000 solution concentrations (*p*<0.05), with no significant differences between 5%, 10%, and the control treatment (*p*>0.05). Germination percentage was significantly higher than the control at 5%, 10%, and 15% PEG-6000 solution concentrations, but significantly lower at 20% PEG-6000 (*p*<0.001). The coefficient of germination rate initially decreased and then increased with increasing PEG-6000 solution concentration, being significantly higher at 15% and 20% PEG-6000 (*p*<0.05), with no significant differences between 5%, 10%, and the control (*p*>0.05). Germination energy was significantly lower under 15% and 20% PEG-6000 solution concentrations compared to the control (*p*<0.01), with the lowest germination energy at 20% PEG-6000 (5.83), which was 34.64% of the control value (16.83). No significant differences were observed among other treatments (*p*>0.05). The highest germination index was 1.50 at 5% PEG-6000, while the lowest was 0.31 at 20% PEG-6000. Significant differences were found in germination index among the all treatments, except for 5% PEG-6000, which did not significantly differ from the control (*p*>0.05). The vigor index decreased with increasing PEG-6000 solution concentration, showing significant differences among the treatments (*p*<0.05) (**Table 3**).

**Table 3:** Effects of simulated drought stress of PEG-6000 on the seed germination of *Lilium concolor* var. *Megalanthum*.

PEG-6000 (%)	DFG	GP	CGR	GE	GI	VI
0	16.60 ± 2.51c	72.50 ± 6.29c	50.35 ± 8.92c	16.83 ± 2.52a	1.44 ± 0.24a	0.05 ± 0.01a
5	14.65 ± 2.01c	95.50 ± 1.37a	49.55 ± 8.06c	16.00 ± 1.67a	1.50 ± 0.20a	0.04 ± 0.01b
10	17.20 ± 2.33c	92.17 ± 2.03ab	49.34 ± 7.82c	15.33 ± 1.38a	1.27 ± 0.17b	0.03 ± 0.00c
15	21.25 ± 2.39b	89.00 ± 2.84b	62.50 ± 7.47b	11.83 ± 1.04b	0.85 ± 0.10c	0.02 ± 0.00d
20	33.10 ± 3.55a	55.50 ± 4.51d	98.02 ± 7.75a	5.83 ± 0.48c	0.31 ± 0.05d	0.01 ± 0.00e

Different lowercase letters in each column represent significant differences at 0.05 levels.

DFG, days to first germination; GP, germination percentage; CGR, coefficient of germination rate; GE, germination energy; GI, germination index; VI, vitality index.

### 3.3 The effect of the interaction between temperature and drought stress on seed germination

**Figure 1:**
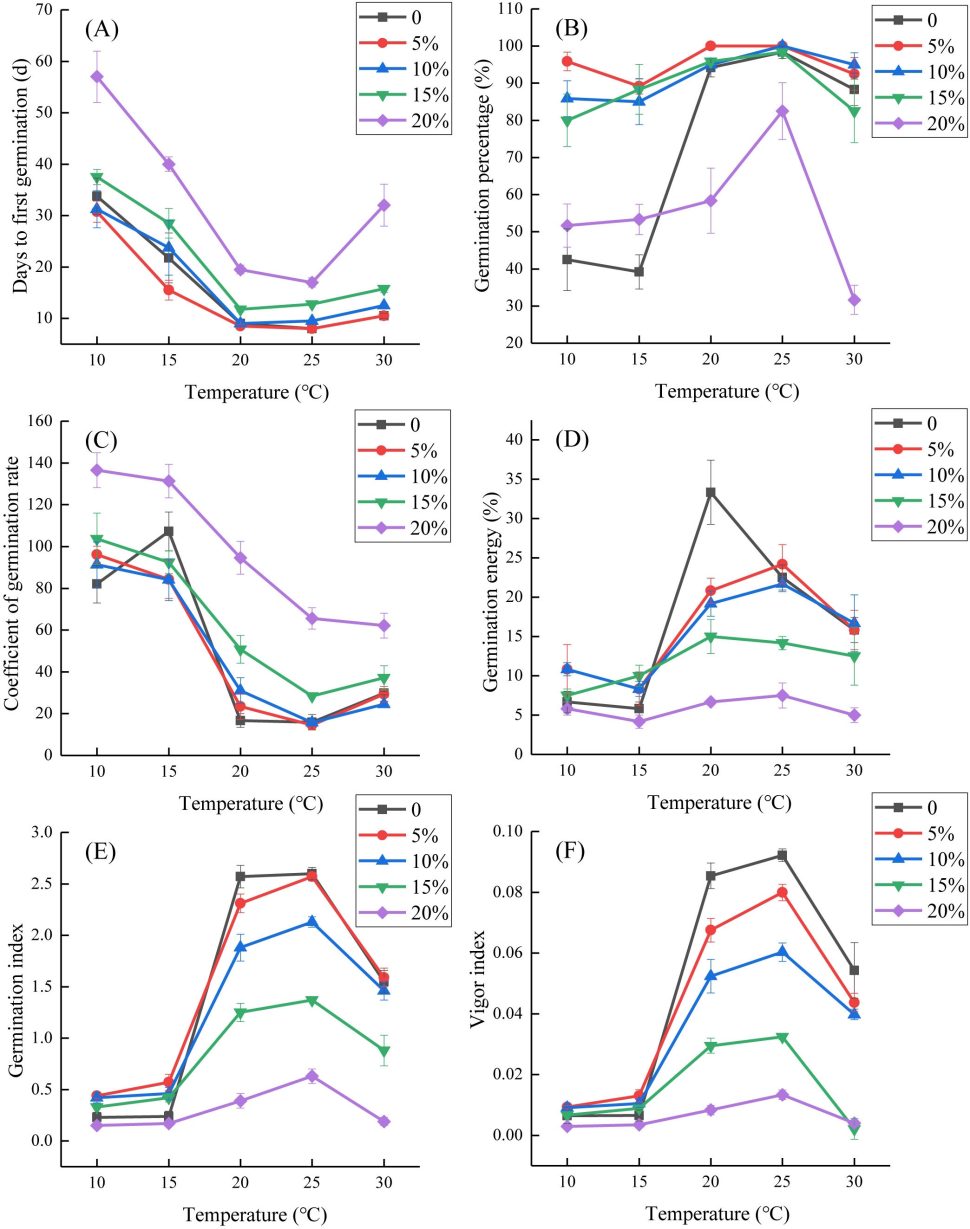
Effects of interactions between temperature and simulated drought stress of PEG-6000 on seed germination of *Lilium concolor* var. *megalanthum*. **(A)** Days to first germination; **(B)** germination percentage; **(C)** coefficient of germination rate; **(D)** germination energy; **(E)** germination index; **(F)** vigor index.

The interaction between temperature and drought stress significantly affected several seed germination parameters of *L. concolor* var. *megalanthum*, including the days to first germination, coefficient of germination rate (*p<*0.05), germination percentage, germination energy, germination index, and vigor index (*p<*0.001) (**Table 1**). The days to first germination of *L. concolor* var. *megalanthum* seeds were lowest under the control treatment and 5% PEG-6000 conditions at 25°C (8 days) and highest under 20% PEG-6000 at 10°C (57 days), resulting in a 49-day delay. The days to first germination significantly increased under 15% PEG-6000 condition at 20°C or 25°C. Additionally, under 20% PEG-6000 condition, the days to first germination significantly increased across all temperature treatments (*p<*0.05) (**Figure 1A**). The highest germination percentage (100%) was observed at 20°C with 5% PEG-6000 and at 25°C with both 5% and 10% PEG-6000, while the lowest germination percentage (31.67%) occurred at 30°C with 20% PEG-6000. At 10°C and 15°C, PEG-6000 concentrations of 5%, 10%, and 15% significantly increased germination percentage (*p<*0.01), but 20% PEG-6000 did not show a significant difference from the control treatment (*p>*0.05). Conversely, at 20°C, 25°C, and 30°C, 20% PEG-6000 significantly reduced germination percentage (*p<*0.05) (**Figure 1B**).

The coefficient of germination rate peaked at 10°C with 20% PEG-6000 (136.54) and was lowest at 25°C with 5% PEG-6000 (14.51). The coefficient of germination rate significantly increased with 15% PEG-6000 at 20°C and 25°C (*p<*0.01) and with 20% PEG-6000 at most temperatures except 15°C (*p<*0.01) (**Figure 1C**). The germination energy was highest in the control treatment at 20°C (33.33%) and decreased with increasing PEG-6000 solution concentrations. It was lowest at 15°C with 20% PEG-6000 (4.17%), with no significant differences at 10°C and 15°C across PEG-6000 concentrations (*p*>0.05). At 20°C, the germination energy was significantly reduced under all PEG-6000 concentrations (*p<*0.05). At 25°C, both 15% and 20% PEG-6000 concentrations significantly reduced germination energy (*p<*0.05). At 30°C, only the 20% PEG-6000 concentration significantly reduced it (*p<*0.05) (**Figure 1D**). The germination index was highest at 25°C in the control treatment (2.59) and lowest at 10°C with 20% PEG-6000 (0.15). At 10°C, it significantly increased with 5% and 10% PEG-6000 (*p<*0.05). At 15°C, only 5% PEG-6000 concentration treatment significantly increased the germination index compared to the control treatment (*p<*0.01). At 20°C and 25°C, 10%, 15%, and 20% PEG-6000 treatments significantly reduced the germination index (*p<*0.01). At 30°C, the germination index was significantly lower under 15% and 20% PEG-6000 treatments compared to the control treatment (*p<*0.01) (**Figure 1E**). The vigor index was highest at 25°C in the control treatment (0.09) and lowest at 10°C and 15°C with 20% PEG-6000 (0). At 15°C, 5% PEG-6000 significantly increased the vigor index (*p<*0.05). At 20°C and 25°C, the vigor index significantly decreased with 5%–20% PEG-6000 (*p<*0.05). At 30°C, it significantly decreased with 10%–20% PEG-6000 (*p<*0.01) (**Figure 1F**).

Correlation analysis revealed that days to first germination were positively correlated with PEG-6000 concentration at all temperatures (*p*<0.01). Germination percentage was negatively correlated with PEG-6000 concentration at 20°C, 25°C, and 30°C (*p*<0.05). The coefficient of germination rate was positively correlated with PEG-6000 concentration at all temperatures except 15°C (*p*<0.01). Germination energy, germination index, and vigor index were negatively correlated with PEG-6000 concentration at 20°C, 25°C, and 30°C (*p*<0.05). At 10°C, only days to first germination and the coefficient of germination rate showed a significant positive correlation with PEG-6000 concentration (*p*<0.01), while other germination parameters had no significant correlations with PEG-6000 (*p*>0.05) (**Table 4**).

**Table 4:** Correlation analysis on the relationship between simulated drought stress of PEG-6000 and germination parameters of *Lilium concolor* var. *megalanthum* seeds at different temperatures (*r-*value).

Temperature (°C)	DFG (d)	GP (%)	CGR	GE (%)	GI	VI
10	0.66**	0.02	0.67**	–0.20	–0.3	–0.42
15	0.68**	0.17	0.34	–0.08	–0.23	–0.35
20	0.81***	–0.63**	0.86***	–0.88***	–0.95***	–0.97***
25	0.91***	–0.52*	0.80***	–0.82***	–0.95***	–0.98***
30	0.78***	–0.69**	0.68**	–0.56*	–0.86***	–0.89***

*p<0.05, **p<0.01, ***p<0.001.

DFG, days to first germination; GP, germination percentage; CGR, coefficient of germination rate; GE, germination energy; GI, germination index; VI, vitality index.

### 3.4 The effects of rehydration on seed germination

**Figure 2:**
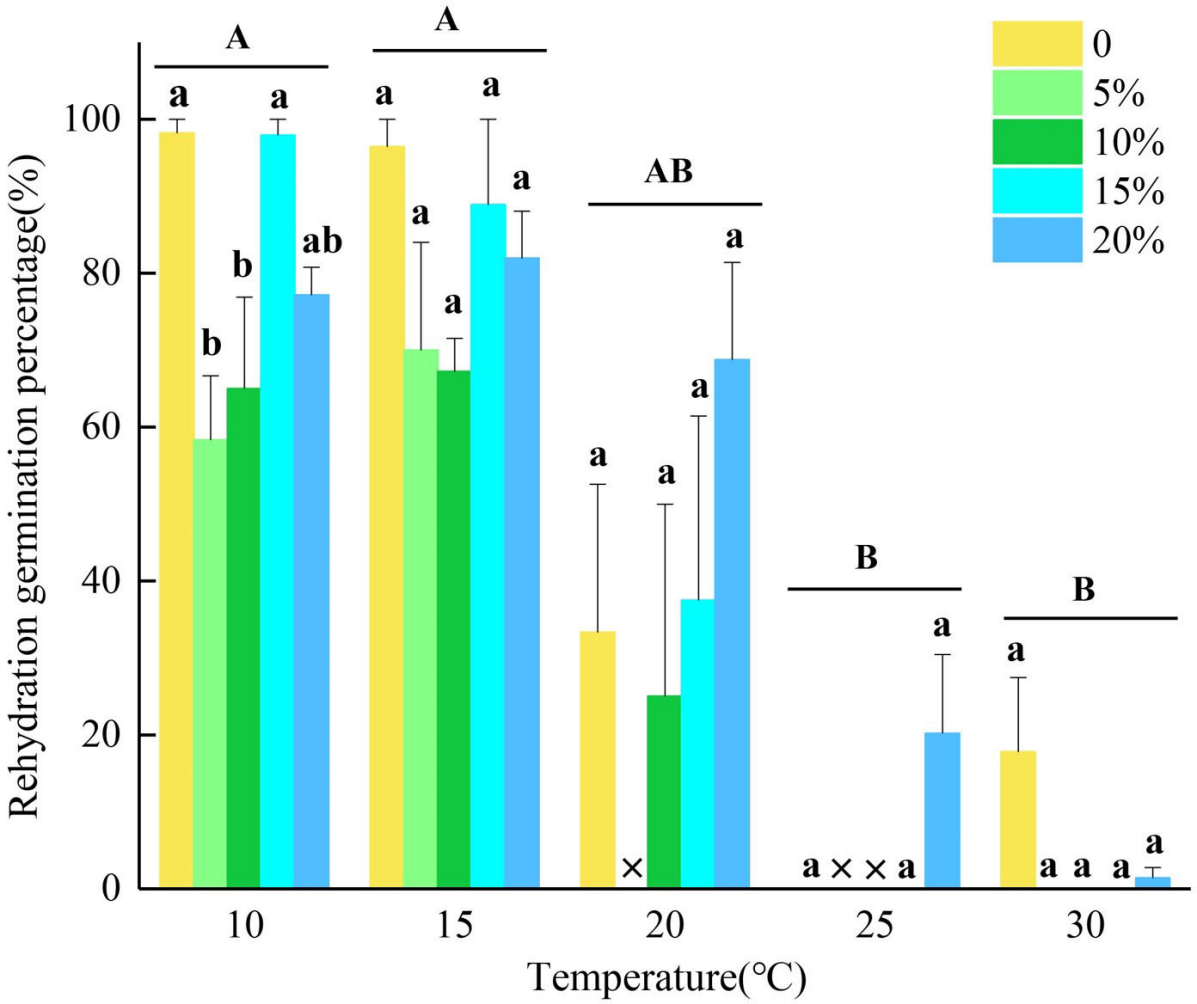
The seed germination percentages of *Lilium concolor* var. *megalanthum* after re-watering under temperature and simulated drought stress of PEG-6000. Different capital letters indicate significant differences in re-watering germination percentages of *Lilium concolor* var. *megalanthum* seeds treated with different temperatures, lowercase letters indicate significant differences in re-watering germination percentages of *Lilium concolor* var. *megalanthum* seeds treated with different concentrations of PEG-6000 solution at the same temperature, and × indicates that the corresponding treatments did not participate in re-watering experiment.

The results showed that seeds that did not germinate at lower temperatures (10°C and 15°C) exhibited rapid and extensive germination after rehydration, with significantly higher germination percentages compared to seeds treated at 25°C and 30°C before rehydration (*p*<0.05). The rehydration germination percentage from the control treatment at 30°C was 17.78% (**Figure 2**). At 25°C, all seeds of *L. concolor* var. *megalanthum* completed germination under drought stress simulated by 5% and 10% PEG-6000 solution concentrations before rehydration experiment, with only two and one seed remaining ungerminated under 0% and 15% PEG-6000 solution concentrations, respectively. These ungerminated seeds did not germinate upon rehydration. The rehydration germination percentage of ungerminated seeds under 20% PEG-6000 treatment was over 20%.

## 4 Discussion

Seed germination is an important mechanism by which plants evade unfavorable environmental conditions over time and space, ensuring population renewal and continuity (Baskin and Baskin, 2004). The germination characteristics of seeds are closely related to the climate and habitat conditions of their natural distribution range. Variations in seed germination characteristics reflect plant adaptation strategies to the environment (Bu et al., 2008). Parameters such as germination percentage and days to first germination serve as pivotal indicators of a seed’s potential transition to the seedling stage (Rosbakh and Poschlod, 2015). The coefficient of germination rate measures the speed of seed germination, with smaller values indicating faster germination and better germination ability within a species. Germination energy and germination index evaluate the rate and uniformity of seed germination, where higher values signify faster germination rates. The vigor index provides insight into the overall germination vigor of seeds. By integrating multiple germination parameters, the germination characteristics of seeds can be comprehensively characterized (Rosbakh and Poschlod, 2015).

### 4.1 The effect of temperature on seed germination

Temperature stands out as a pivotal factor affecting seed germination (Verma et al., 2010). For *L. concolor* var. *megalanthum* seeds, the earliest days to first germination and highest germination energy occurred at 20°C and 25°C. The germination percentage, coefficient of germination rate, germination index, and vigor index were all maximized at 25°C, indicating that 25°C is the optimum temperature for *L. concolor* var. *megalanthum* seed germination. Extremes of temperature, whether too high or too low, adversely affect seed germination. Specifically, low temperatures reduce seed vigor and inhibit seed germination (Tanaka-Oda et al., 2009). *L. concolor* var. *megalanthum* seeds mature and disperse by late September, coinciding with soil surface temperature in peatlands dropping below 15°C. This condition delays days to first germination and reduces seed germination percentage. From November to April, temperatures plunge below 0°C, and wetland is covered by snow. Even if a few seeds germinate during this period, subsequent survival of their seedlings is hindered by prolonged exposure to low temperature. Similarly, low temperature in early spring delays germination, impeding *L. concolor* var. *megalanthum* seedlings from establishing themselves and limiting population renewal rates. This may explain the sparse population of *L. concolor* var. *megalanthum* in the wild. However, some ungerminated *L. concolor* var. *megalanthum* seeds enter dormancy, enabling them to evade the challenge of seedlings survival in low temperatures post-germination. They await suitable conditions to germinate, establishing roots before high temperature in summer, significantly enhancing seedling survival prospects and population establishment.

### 4.2 The effect of drought on seed germination

Under normal conditions, drought negatively affects seed germination, leading to reduced germination and seedling survival rates and stunted growth and development of plant seedlings (Campos et al., 2020). However, studies indicate that moderate drought stress can enhance seed germination in some plant species. For instance, Tang et al. (2019) used three concentrations of PEG-6000 solution to simulate varying drought stress levels and studied the seed germination of seven genotypes of *Hibiscus cannabinus* to assess their drought resistance. Their findings revealed that the 10% PEG-6000 solution treatment promoted seed germination compared to both the CK and the 20% PEG-6000 solution treatment.

Similarly, the mild drought stress induced by PEG-6000 solution concentrations (5% and 10%) promoted the germination of *Apocynum venetum* seeds, with higher germination percentages, germination indices, and vigor indices compared to the control treatment, and promoting the onset of germination. However, as the concentration of PEG-6000 solution increased, germination was inhibited, with the inhibitory effect becoming more pronounced with higher concentrations, leading to a greater decline in germination parameters (Xu et al., 2015). Consistent with the above study, in this research, PEG-6000 solution concentrations of 5%, 10%, and 15% all increased the germination percentage of *L. concolor* var. *megalanthum* seeds to varying degrees. Under the 15% PEG-6000 solution concentration, the days to first germination were delayed, the coefficient of germination rate increased, and the germination energy, germination index and vigor index decreased. However, the 5% PEG-6000 solution concentration helped to enhance the germination index. Overall, the 5% PEG-6000 solution concentration-simulated drought intensity significantly promoted the germination of *L. concolor* var. *megalanthum* seeds and optimized various germination parameters. Although the 10% and 15% PEG-6000 solution concentrations increased the germination percentage, they delayed the days to first germination and reduced the germination energy, germination index, and vigor index. The 20% PEG-6000 solution concentrations strongly inhibited the germination of *L. concolor* var. *megalanthum* seeds. This result may be due to the slow and insufficient water absorption by seeds under extremely low water potential conditions, which inhibits respiration and energy production, leading to insufficient energy to initiate the germination process, thus suppressing seed germination (Mayer and Poljakoff-Mayber, 1989). *L. concolor* var. *megalanthum* seeds exhibit strong drought tolerance compared to *Foeniculum vulgare* seeds, which cannot tolerate −0.3 MPa PEG-6000 osmotic potential (Stefanello et al., 2006). The 20% PEG-6000 solution concentration used in this study (with an osmotic potential of −0.6 MPa) has not yet reached the critical PEG-6000 concentration tolerated by *L. concolor* var. *megalanthum*.

In its natural habitat, *L. concolor* var. *megalanthum* grows on hummocks of *Carex* spp., alongside various other species such as *Lythrum salicaria*, *Geranium wilfordii*, *Lysimachia davurica*, and *Patrinia scabiosaefolia*. The presence of litter around the seeds can hinder their contact with soil moisture, thereby affecting both germination and seedling survival (Kubo et al., 2004). The seeds of *L. concolor* var. *megalanthum* are extremely lightweight, making it challenging for them to penetrate through thick accumulations of wetland plant litter and reach the soil. This difficulty in reaching soil moisture directly impacts seed germination, potentially posing a significant obstacle to natural population regeneration.

### 4.3 The combined effect of temperature and drought on seed germination

In the combined conditions of 25°C temperature and 5% PEG-6000 solution concentration, *L. concolor* var. *megalanthum* seeds exhibited optimal germination parameters. The sensitivity of seed germination to drought stress is influenced by the germination temperature (Menge et al., 2016). Studies have shown that drought stress significantly inhibits the germination of *Helianthus annuus* seeds, especially when the optimum temperature exceeds 20°C (Toscano et al., 2017). Similarly, higher temperatures exacerbate the inhibitory effects of drought stress on seed germination of *Chloris virgata* (Lin et al., 2016). Moreover, the combined impact of elevated temperature and reduced water potential sharply reduces the germination percentage of *Pinus yunnanensis* seeds (Gao et al., 2021). In this study, at temperatures of 10°C and 15°C, the germination percentage, germination index, and vigor index increased with 5%, 10%, and 15% PEG-6000 solution concentration. However, under 20% PEG-6000 solution concentration, there was a significant delay in the days to first germination, and the coefficient of germination rate increased. At temperatures of 20°C, 25°C, and 30°C, conditions with 10%, 15%, and 20% PEG-6000 solution concentrations resulted in decreased germination index and vigor index. Specifically, the 15% PEG-6000 solution concentration delayed the days to first germination, reduced germination energy, and increased the coefficient of germination rate. The 20% PEG-6000 solution concentration led to decreased germination percentage, germination index and vigor index, delayed days to first germination, and increased the coefficient of germination rate.

These results indicate that seeds exhibit strong tolerance to drought stress simulated by PEG-6000 solution under low temperatures, and moderate drought can actually promote seed germination. Seeds of *L. concolor* var. *megalanthum* exhibit heightened sensitivity to drought stress under higher temperature, intensifying the inhibitory effects of drought stress on seed germination. This is consistent with the results that PEG stress can promote the germination of *Henophyton deserti* seeds at lower temperature but inhibits germination at higher temperature (Gorai et al., 2014). In response to fluctuating or unpredictable environments, seeds employ different strategies. They may germinate rapidly to enhance their competitive advantage in arid conditions by extending the growth time of seedlings after germination. Alternatively, seeds may employ delayed germination as a buffer strategy to avoid germinating and reproducing within the same year, thereby reducing reproductive failure risks across their distribution area (Rice and Dyer, 2001; Zeng et al., 2010; Duncan et al., 2019). In spring’s lower temperatures, mild drought stress can actually benefit the germination of *L. concolor* var. *megalanthum* seeds, allowing ample time for root establishment before the onset of hotter summer temperatures, thereby enhancing seedlings survival. However, as summer temperature rises, these seeds become more sensitive to drought stress, potentially leading them to enter dormancy and form a soil seed bank. This dormancy strategy serves a dual purpose: it protects seedlings from challenging conditions of high temperatures and drought immediately after germination while also providing an opportunity to await more favorable conditions for subsequent germination attempts. We germinated *L. concolor* var. *megalanthum* seeds that had been stored for 3 years and found that their vigor could persist beyond 3 years. This extended seed vigor exemplifies an adaptive germination mechanism that allows *L. concolor* var. *megalanthum* to effectively respond to environmental changes over the course of its long-term reproductive process.

### 4.4 Seed re-germination after stress relief

Environmental factors can indirectly influence seed germination characteristics by altering intrinsic seed properties, such as seed morphology and nutrient content (Wu et al., 2018). Seeds that do not germinate under certain temperature and drought stress conditions during initial germination attempts may exhibit resumed germination when transferred to non-stressful conditions (Miranda et al., 2014; Hu et al., 2015; Gu et al., 2018). Previous studies have showed that seeds of *Seriphidium transiliense* and *Salsola imbricata*, which could not germinate under conditions of low water potential and high temperature, resumed germination immediately upon transfer to distilled water, indicating that most of these seeds can still germinate after alleviating the effects of low water potential (Elnaggar et al., 2018; Chen et al., 2020). Based on the rehydration germination percentages of *L. concolor* var. *megalanthum* seeds transferred to 25°C after treatment with temperature and PEG-6000 solution stress, seeds treated with 5% and 10% PEG-6000 solutions exhibited rehydration germination percentage lower than those treated with 0%, 15%, and 20% PEG-6000 solution. This could be attributed to the fact that seeds treated with 5% and 10% PEG-6000 solutions promoted germination of some seeds, while in others, despite not germinating, internal physiological and biochemical changes occurred that depleted the seeds’ internal storage nutrients, thereby preventing germination upon rehydration. However, most of the seeds treated with 0%, 15%, and 20% PEG-6000 solution exhibited mostly complete inhibition, preserving internal seed nutrients intact. Upon transfer to suitable temperatures and relief from drought stress, these seeds germinated immediately.

## 5 Conclusion

This study investigated the effects of temperature, drought stress, and their interaction on the germination of *L. concolor* var. *megalanthum* seeds. The results showed that conditions simulating drought stress at 25°C with a 5% PEG-6000 solution were most conducive to seed germination. Seeds of *L. concolor* var. *megalanthum* exhibited the highest tolerance to PEG-6000 simulated drought stress at 10°C and 15°C. Moderate drought stress under low temperature conditions promoted seed germination. Conversely, seeds were more sensitive to drought stress at high temperatures, with increased temperature intensifying the inhibitory effects of drought stress on seed germination of *L. concolor* var. *megalanthum*. Following the relief of stress conditions, the seeds of *L. concolor* var. *megalanthum* resumed germination. The germination of *L. concolor* var. *megalanthum* seeds demonstrates a certain selective adaptability to temperature and moisture conditions, likely evolved as an ecological adaptation strategy in long-term wetland environments.

## Data Availability

The original contributions presented in the study are included in the article/supplementary material. Further inquiries can be directed to the corresponding authors.
